# Comparative Analysis of Clavicular Hook Plate Versus Distal Radius Volar Plate With Coracoclavicular Augmentation for Neer’s Type 2b Lateral Clavicle Fractures: A Prospective Study

**DOI:** 10.7759/cureus.22969

**Published:** 2022-03-08

**Authors:** Ajinkya R Bandebuche, Abhishek K Rai, Dixit Bansal, Devanshu Gupta, Ajay Naidu

**Affiliations:** 1 Orthopaedics, Lokmanya Tilak Municipal Medical College and General Hospital (LTMGH), Mumbai, IND; 2 Orthopaedics, Seth Gordhandas Sunderdas Medical College (GSMC) and King Edward Memorial (KEM) Hospital, Mumbai, IND; 3 Orthopedics, Government Medical College, Amritsar, Amritsar, IND

**Keywords:** constant score, dash score, vas score, lateral clavicle fractures, coracoclavicular augmentation, distal radius plate, clavicular hook plate

## Abstract

Background

Clavicle fractures are common injuries in the adult population. The commonest site of fracture in the clavicle is the mid-shaft followed by the lateral end fracture. The anatomy and biomechanics of the lateral end clavicle make it prone to be unstable. Conservative management usually fails due to the deforming forces.

Aim

Our study evaluates pain relief, functional outcome, and the union rate in unstable lateral end clavicle fracture fixed by two different modalities of operative management, namely clavicular hook plate fixation and distal radius volar plate fixation.

Materials and method

A total of 60 patients with the unstable lateral end of clavicle fracture were evaluated in this study at a single tertiary care center between August 2015 and September 2021. Half of the patients (30 patients) were managed by open reduction and internal fixation with clavicular hook plate. The remaining half (30 patients) underwent open reduction and internal fixation by distal radius volar plate supplemented with coracoclavicular fixation. All patients were followed up for a mean duration of 20 months. The functional outcome was assessed at regular intervals by Constant score and Disability of the Arm, Shoulder and the Hand (DASH) score for a period of one year.

Result

There was significant pain relief and improvement in the functional status of patients. The pain relief was significant in the group managed by distal radius volar plate. The decrease in DASH score and increase in Constant score suggests better functional outcomes in these patients.

Conclusion

Our study highlights the fact that the distal radius volar plate is an excellent alternative to the hook plate in the treatment of unstable lateral third clavicle fractures. The decrease in pain and improved functional outcome stresses the fact that the volar locking plate is the recent most advancement in the fracture fixation of Neer’s type ll fractures. The distal radius volar plate is the recent internal fixation technique to manage unstable lateral end clavicle fractures.

## Introduction

Fractures of the clavicle are common injuries of adults, accounting for about 2.6 to 4% of all injuries. They are usually caused by either a direct anterior blow or by a fall on the outstretched hand [1]. Clavicle fractures are categorized into proximal, mid-shaft, and distal fractures. The commonest site of fracture in the clavicle is the mid-shaft followed by the lateral end fracture. Of these lateral end clavicle fractures, 40-52% are displaced fractures which are unstable due to the displacing forces acting on the fracture fragments: an inferior force on the lateral clavicle fracture fragment and an anterosuperior force on the medial clavicle fragment [2].

The lateral fractured fragment is small and thus, poses difficulty to get an anatomical reduction resulting in instability of the lateral clavicle fractures. Non-operative methods are associated with high rates of non-union. The operative treatment includes internal fixation by various modalities. Osteosynthesis with an anatomical locking plate, hook plate, and fixation with distal radius locking plate are the recently available options [3]. There is a paucity of literature regarding the best management option for these unstable fractures. This study highlights the benefit of distal radius volar plate over clavicular hook plate in the management of unstable, displaced lateral end clavicle fractures in terms of pain relief, union rate, and functional outcome. The functional outcome was assessed by Constant score and Disability of the Arm, Shoulder and the Hand (DASH) score [4].

## Materials and methods

The prospective study was conducted at the department of orthopedics in a tertiary care center after getting approval from Institutional Review Board (IRB) before starting the study (Institutional Review Board, dated 24th June 2015; approval number: IRB/PS/2015/22). The selection criteria include all unilateral cases of Neer’s type II unstable fractures of lateral end clavicle managed by open reduction and internal fixation. The exclusion criteria include undisplaced stable fractures (Neer’s type I) and pathological fractures. After clinico-radiological examination of the patients, the acromioclavicular joint was temporarily immobilized with chest-arm strapping and an arm sling. This study was a single tertiary care center, prospective study, which included all unilateral cases of Neer’s type II unstable fractures of lateral end clavicle between August 2015 and September 2021. Sixty patients (42 males, 18 females) were included in the study. The mean age was 41.4 years (range: 19-68 years). Half of the patients (30 patients) were managed by open reduction and internal fixation with clavicular hook plate. The remaining half (30 patients) underwent open reduction and internal fixation by distal radius volar plate supplemented with coracoclavicular fixation. The coracoclavicular fixation options were determined by the fracture pattern and the coracoclavicular displacement. The coracoclavicular fixation was done either by mersilene tape and endobutton or by coracoclavicular screw. Both hook plate and the distal radius volar plate were made of stainless steel and manufactured by the local implant distributor. The depth of the hook in all hook plates was constant i.e., 15mm. The post-operative physiotherapy and mobilization protocols were the same for all patients. The shoulder joint was immobilized in an arm sling post-operatively for a week. The pendular movements and passive range of motion as per pain tolerance were started after a week. All patients were assessed clinico-radiologically at regular weekly follow-up till union at the fracture site. The clinical criteria to define fracture union was absent of significant pain or tenderness on palpation and weight-bearing. The radiological criteria were bridging of the fracture at three cortices and obliteration of the fracture line. The patients were instructed to start active range of motion of shoulder joint and to do their daily routine activities after fracture union. Sports activities were limited for 12 weeks postoperatively. The pain was assessed by the Visual Analog Scale (VAS) score. The functional outcome was assessed by Constant score and Disability of the Arm, Shoulder and the Hand (DASH) score. The VAS score is a unidimensional pain measurement scale, which ranges from 0 to 10. The VAS score is evaluated as 0 (no pain), one to three (mild pain), four to six (moderate pain), and seven to 10 (severe pain). The Constant score is a 100-point scale composed of parameters that define the level of pain and the ability to carry out the daily activities of the patient, consisting of four subscales: pain (15 points), activities of daily living (20 points), strength (25 points) and range of motion (40 points). The DASH questionnaire is a 30-item questionnaire that looks at the ability of a patient to perform certain upper extremity activities. The scores for all items are then used to calculate a scale score ranging from 0 (no disability) to 100 (most severe disability). The patients were followed up postoperatively every week till union and thereafter routinely at six weeks, three months, six months, and 12 months for clinical evaluation and radiographs.

Data analysis

The data was collected, compiled, and analyzed. The descriptive data (mean and standard deviation) were calculated for continuous variables. Frequencies and percentages were calculated for categorical variables. Association between variables was analyzed by Chi-Square test for categorical Variables. An unpaired t-test was used to compare the mean of quantitative variables between the two study groups.

Surgical technique for clavicular hook plate

**Figure 1:**
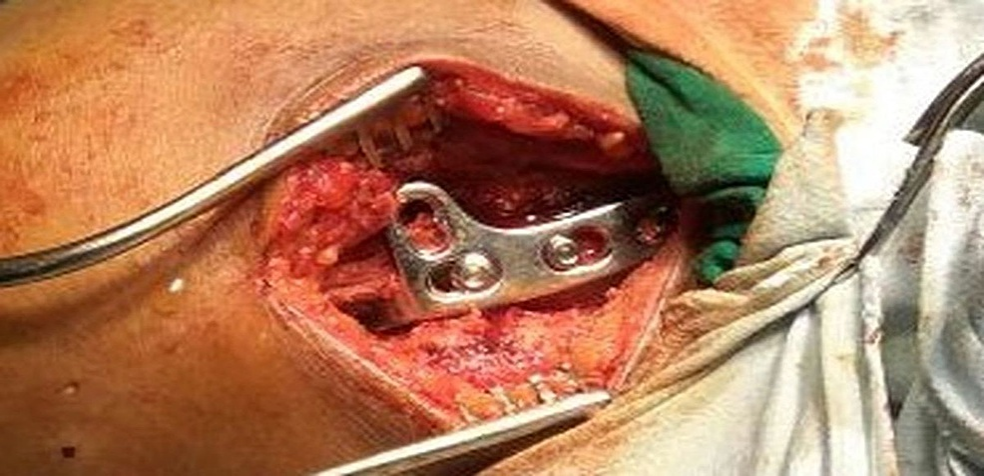
Intra-operative image of lateral end clavicle fracture managed by clavicular hook plate.

**Figure 2:**
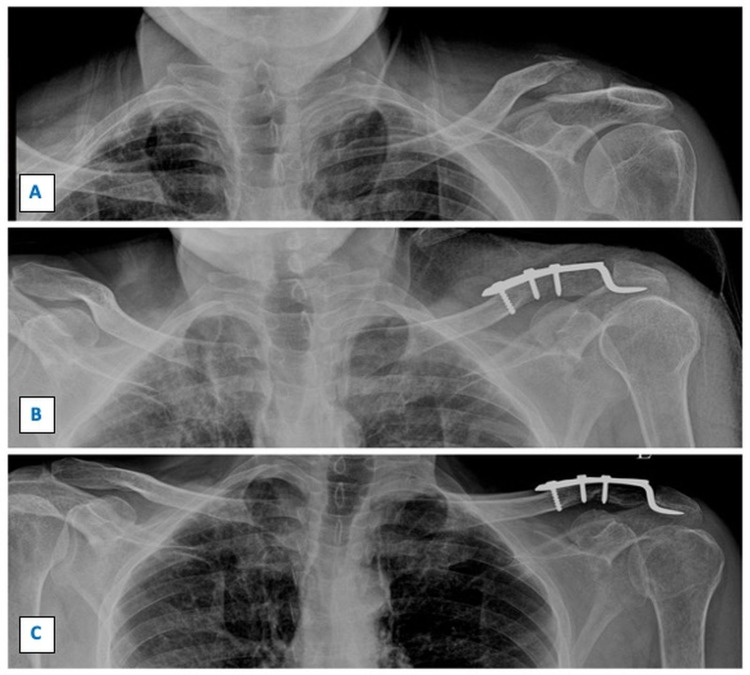
Sequential radiographs showing fracture of left lateral end clavicle managed by clavicular hook plate. Figure 2A shows the preoperative radiograph with displaced left lateral end clavicle fracture. Figure 2B shows immediate postoperative radiograph managed by clavicular hook plate. Figure 2C shows the radiograph at the one-year follow-up with the union of the fracture site.

The patients were operated in beach chair position under general anesthesia with a 6-8 cm sagittal incision medial to the acromioclavicular joint. Full-thickness skin flaps were prepared until the bone for better closure clavicle. The fracture was reduced and the fragments were temporarily fixed with Kirschner wires or lag screws. Without opening the acromioclavicular joint, the location of the joint was marked with a needle and confirmed with fluoroscopy. The soft tissue dorsal to the acromioclavicular joint was dissected and prepared for the placement of the hook of the plate. The hook depth of 15 mm was fixed for all plates. The hook of hook plate was passed below the acromion (Figure 1). Before definitive fixation, the position of the plate and shoulder motion was confirmed under fluoroscopy (Figure 2).

Surgical technique for distal radius volar plate

**Figure 3:**
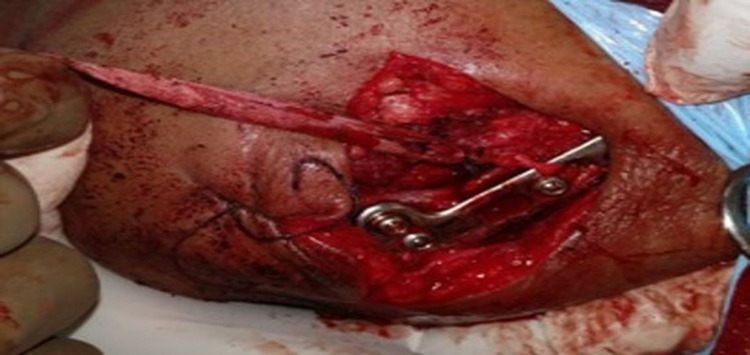
Intra-operative image of lateral end clavicle fracture managed by distal radius volar plate and coracoclavicular fixation with mersilene tape and endobutton.

**Figure 4:**
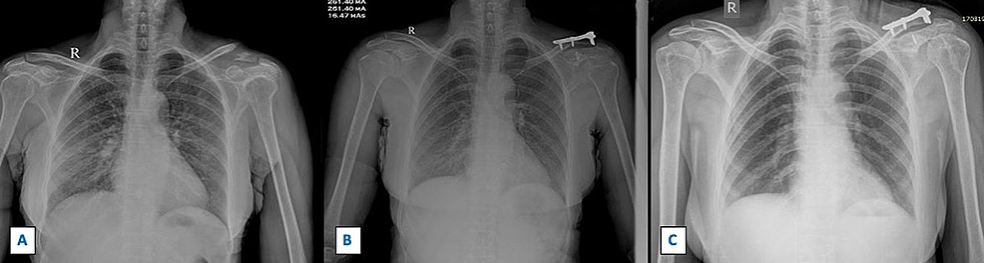
Sequential radiographs showing fracture of left lateral end clavicle managed by distal radius volar plate with coracoclavicular fixation. Figure 4A shows the pre-operative radiograph with displaced left lateral end clavicle fracture. Figure 4B shows immediate post-operative radiograph managed by distal radius volar plate and coracoclavicular fixation with mersilene tape and endobutton. Figure 4C shows the radiograph at one-year follow-up with the union of the fracture site.

The patients were operated in beach chair position under general anesthesia with a sabre-cut approach medial to the acromioclavicular joint to visualize the fracture site and the coracoid process. The deltoid fascia was incised in line with the lateral clavicle. Coraco-clavicular fixation was done by 4 mm cancellous cannulated( CC) screw in young patients with good bone quality while in patients with inadequate purchase of CC screw, endobutton with mersilene tape was used. The fracture was reduced under direct visualization and intraoperative fluoroscopy control. A distal radius volar locking plate was applied for internal fixation. The sutures were then transferred around the clavicle and over the plate (Figure 3). The delto-trapezoid fascia was reconstructed with absorbable sutures and the wound was closed in layers (Figure 4).

## Results

A total of sixty patients who presented to our outpatient department (OPD) during the study were included in the study. There were 42 males (70%) and 18 females (30%). The mean age was 41.4 years (range: 19-68 years). The mean follow-up was 20.6 months (12-36 months). A total of 60 patients were operated on by two different modalities of internal fixation. Half of the patients (30 patients) were managed by open reduction and internal fixation with clavicular hook plate. The remaining half (30 patients) underwent open reduction and internal fixation by distal radius volar plate supplemented with coracoclavicular fixation. The patients were followed up postoperatively every week till union and thereafter routinely at six weeks, three months, six months, and 12 months for clinical evaluation and radiographs. The clinical evaluation includes VAS score for pain relief and for functional improvement, Constant score, and DASH score were used. The union of the fracture site was assessed radiologically by serial x-rays. The decrease in the VAS score of the patients operated by distal radius volar plate was statistically significant at three months, six months, and twelve months as compared to the group who were managed by clavicular hook plate (Table 1). The pain relief was significant in the group managed by distal radius volar plate. The decrease in the DASH score of the patients operated by distal radius volar plate was statistically significant at three months, six months, and twelve months as compared to the group who were managed by clavicular hook plate, suggesting a better functional outcome (Table 2). The Constant score increased significantly at three months, six months, and one year in the patients who were operated by distal radius volar plate (Table 3). The increase in Constant score suggests better functional outcomes in these patients. The mean union rate for distal radius volar plate and clavicular hook plate was 8.4 weeks (range: 6-12 weeks) and 11.2 weeks (range: 8-18 weeks) respectively (Table 4). Of all the patients operated by distal radius volar plate, one patient reported mal-union and two patients had postoperative shoulder stiffness at one year follow up. In the other group of patients managed by clavicular hook plate, two patients reported mal-union, five had impingement, three had shoulder stiffness, and one case of subacromial osteolysis (Table 5). At a mean follow-up of 20 months, none of the patients reported infection, non-union, or procedure-related neurovascular injury.

**Table 1:** Comparison of VAS score between two different modalities of fixation; Clavicular hook plate and Distal radius volar plate. * Values are significant; p < 0.05 VAS: visual analog scale

Operative fixation modality	VAS score			
	6 weeks	3 months	6 months	12 months
Clavicular hook plate	6.3 ± 1.2	3.2 ± 0.65	2.3 ± 0.32	1.6 ± 0.18
Distal radius volar plate	6.6 ± 1.4	2.6* ± 0.36	1.6* ± 0.18	0.4* ± 0.12
p value(<0.05)	0.062	<0.01	<0.01	<0.01

**Table 2:** Comparison of DASH score between two different modalities of fixation; Clavicular hook plate and Distal radius volar plate. * Values are significant; p < 0.05 DASH: Disability of the Arm, Shoulder and the Hand

Operative fixation modality	DASH score			
	6 weeks	3 months	6 months	12 months
Clavicular hook plate	12.4 ± 1.7	8.6 ± 1.24	5.1 ± 0.86	1.8 ± 0.16
Distal radius volar plate	10.6 ± 1.8	3.6* ± 0.42	2.2* ± 0.14	0.6* ± 0.1
p value(<0.05)	0.085	<0.01	<0.01	<0.01

**Table 3:** Comparison of Constant score between two different modalities of fixation; clavicular hook plate and distal radius volar plate. * Values are significant; p < 0.05

Operative fixation modality	Constant score			
	6 weeks	3 months	6 months	12 months
Clavicular hook plate	75.2 ± 2.6	78.6 ± 2.2	82.1 ± 1.66	86.7 ± 1.24
Distal radius volar plate	78.7 ± 2.8	90.5* ± 1.74	94.2* ± 1.26	98.1* ± 0.42
p value(<0.05)	0.144	<0.01	<0.01	<0.01

**Table 4:** Comparison of mean fracture union rate between two different modalities of fixation; clavicular hook plate and distal radius volar plate

Operative fixation modality	Fracture Union(weeks)	
	Mean(weeks)	Range(weeks)
Clavicular hook plate	11.2	6-12
Distal radius volar plate	8.4	8-18

**Table 5:** Comparison of various postoperative complications between two different modalities of fixation; clavicular hook plate and Distal radius volar plate

Post-Operative Complications	Clavicular hook plate( Number of patients out 0f total 30)	Distal radius volar plate( Number of patients out of total 30)
Infection	0	0
Malunion	2(6.67%)	1(3.33%)
Non Union	0	0
Impingement	5 (16.67%)	0
Shoulder Stiffness	3 (10%)	2(6.67%)
Subacromial Osteolysis	1 (3.33%)	0

## Discussion

Clavicle fractures are common injuries in the adult population. But the displaced lateral end clavicle fracture is an uncommon finding. Although 15% of all clavicle fractures consist of lateral clavicle fractures, only a third of these fractures are displaced and unstable. The clavicle fractures were generally treated by conservative means. The distal fragment in lateral end clavicle fractures is small and the deforming forces are great, the conservative management fails as it is associated with the significant incidence of complications like malunion, non-union and joint stiffness. An increase in the functional demand of patients in recent times has changed the statistics of operative management. Operative management offers stable fixation and an early range of motion. 

This study was conducted to evaluate and compare pain relief, functional outcome, and the union rate in lateral end clavicle fracture fixed by two different modalities of operative management, namely clavicular hook plate fixation and distal radius volar plate fixation. A total of 60 patients with unstable lateral end of clavicle fracture attending our tertiary care hospital were evaluated in this study. Half of the patients (30 patients) were managed by open reduction and internal fixation with clavicular hook plate. The remaining half (30 patients) underwent open reduction and internal fixation by distal radius volar plate supplemented with coracoclavicular fixation. All patients were followed up with a mean duration of 20 months.

**Figure 5:**
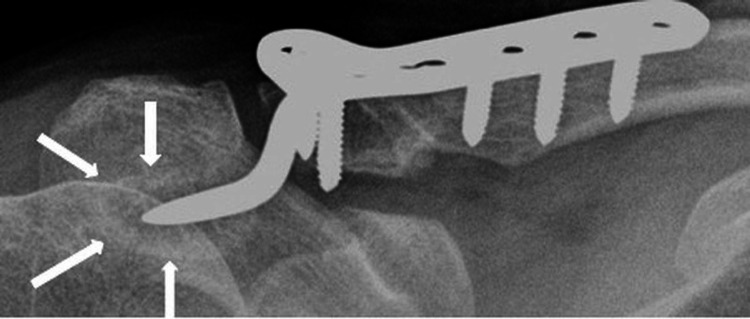
Radiograph showing subacromial osteolysis (arrows) after clavicular hook plate fixation.

**Figure 6:**
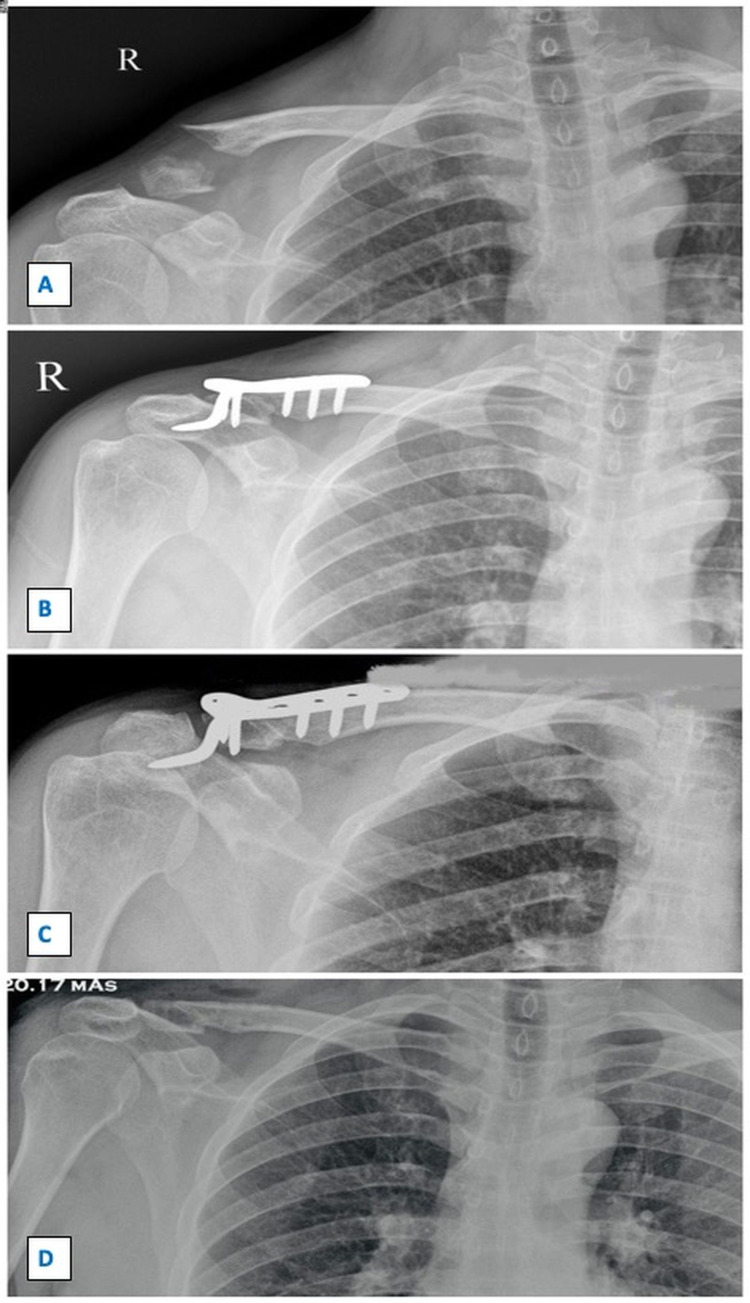
Sequential radiographs showing fracture of right lateral end clavicle managed by clavicular hook plate. Figure 6A shows the pre-operative radiograph with displaced right lateral end clavicle fracture. Figure 6B shows immediate post-operative radiograph managed by clavicular hook plate. Figure 6C shows united fracture at three-month follow-up with subacromial osteolysis and impingement. Figure 6D shows the radiograph after clavicular hook plate removal with the complete union at the fracture site.

**Figure 7:**
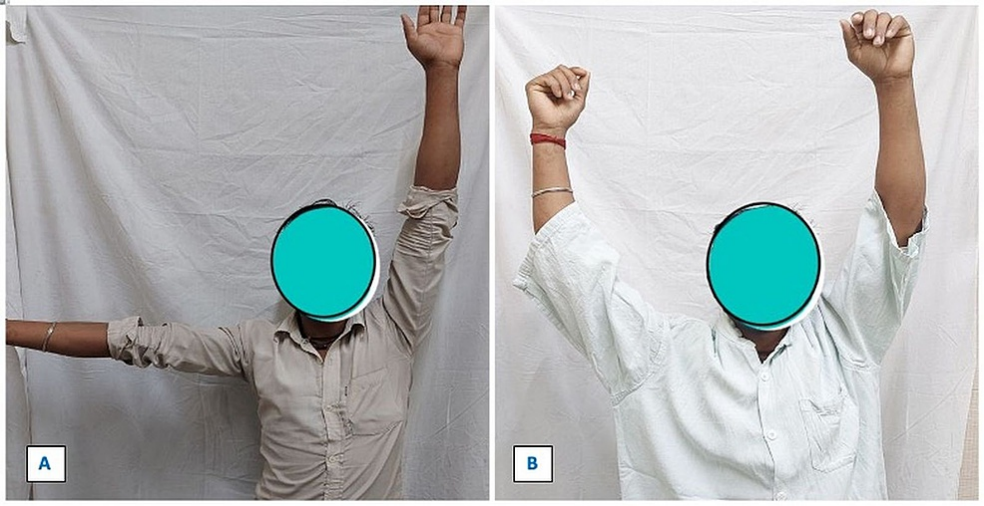
Clinical picture showing the abduction of the right shoulder before and after clavicular hook plate removal. Figure 7A shows restricted abduction of the right shoulder due to impingement by hook plate. Figure 7B shows the full range of movement after the removal of the clavicular hook plate.

The hook plate is a reliable surgical modality for decades for lateral end clavicle fractures. Saeed et al. reviewed 24 original articles in their meta-analysis and concluded that hook plate is as effective as tension band wiring or coracoclavicular fixation in regards to functional activity [5]. The subacromial hook provides leverage to the distal fragment and keeps it in line with the superiorly displaced medial fragment. Constant contact with the subacromial hook predisposes to the inflammation of subacromion leading to osteolysis of acromion. Among all the complications, subacromial osteolysis is the most common. In our study, one patient managed with clavicular hook plate had subacromial osteolysis noted at three months follow up (Figure 5). The bony union had occurred and thus, we planned for the removal of the implant (Figure 6). The functional outcome improved after the implant removal (Figure 7).

The distal radius volar plate fits anatomically to the inflated contour of the lateral end clavicle. The 3.5 mm hole of the plate approximates in size of the lateral clavicle. The volar plate is strong, and angular which facilitates fixation of the small distal fragment. The low profile of the plate allows better skin closure. There are less than ten studies in literature emphasizing the importance of these plates in Neer’s type ll fractures. Chao et al. managed six cases of lateral end clavicle fractures with volar plate [6]. The functional and radiological outcome achieved was satisfactory to the patient with no complications. Ashraf et al. studied the results of internal fixation with distal radius volar plate on fifteen patients [7]. Thirteen patients achieved full range of motion and the other two had minor limitations. Bulent et al. evaluated the results of surgical treatment of lateral end clavicle fractures by distal radius locking plate [8]. All patients achieved full range at six weeks follow up. The functional outcome was assessed by a modified shoulder rating scale and the Constant score. They concluded that clinical results were excellent with no complications reported. Erik et al. reviewed the outcome of unstable lateral end clavicle fracture with a superiorly placed distal radius locking plate [9]. The functional outcome was assessed by Shoulder Pain And Disability Index (SPADI), DASH score, and Constant score. They concluded that the volar plate supplemented with tightrope augmentation preserves excellent shoulder range. A detailed review of PubMed highlights only one study which compared the functional outcome of hook plate versus distal radius volar plate. Hong et al. compared the clinical outcomes of hook plate and distal radius T-plate for fixation of lateral end clavicle [10]. The study included forty-two patients, of whom, 23 were managed by hook plate and the rest 19 by volar plate. The patients were evaluated on the basis of the University of California, Los Angeles (UCLA) Shoulder rating scale. They concluded that the distal radius volar plate yielded excellent results as compared to the hook plate.

**Figure 8:**
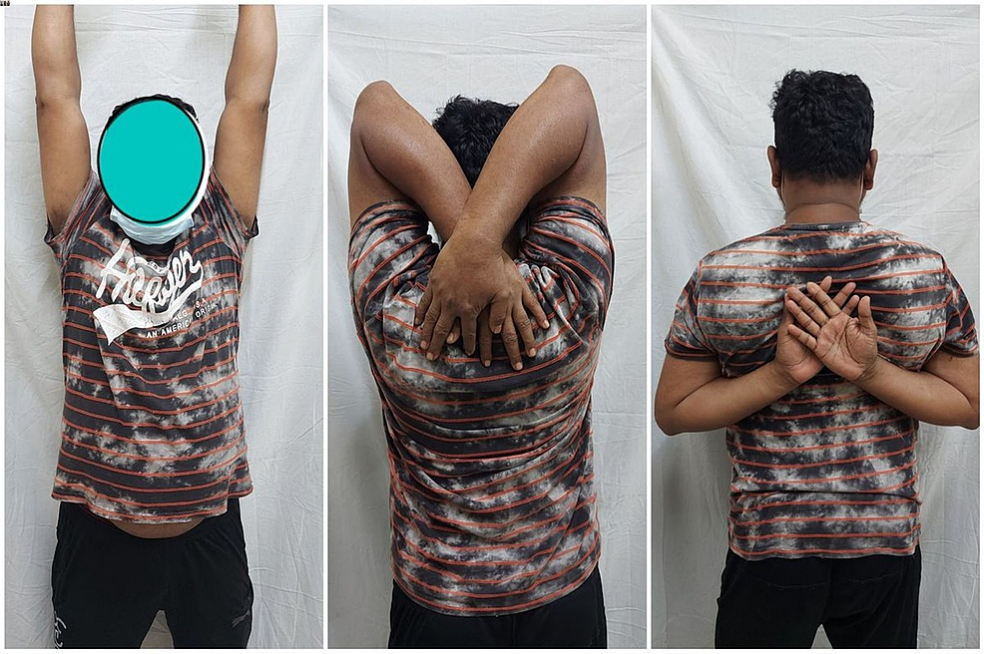
Clinical picture of a 35-year-old male showing the full range of left shoulder movement at one-year follow-up.

**Figure 9:**
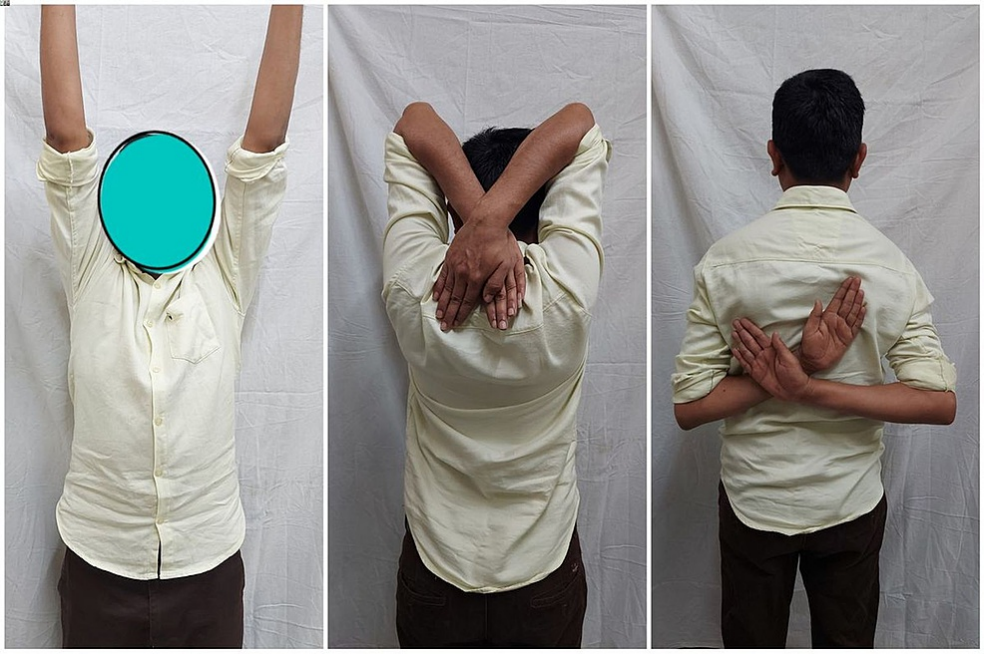
Clinical picture of a 28-year-old male showing the full range of left shoulder movement at one-year follow-up.

**Figure 10:**
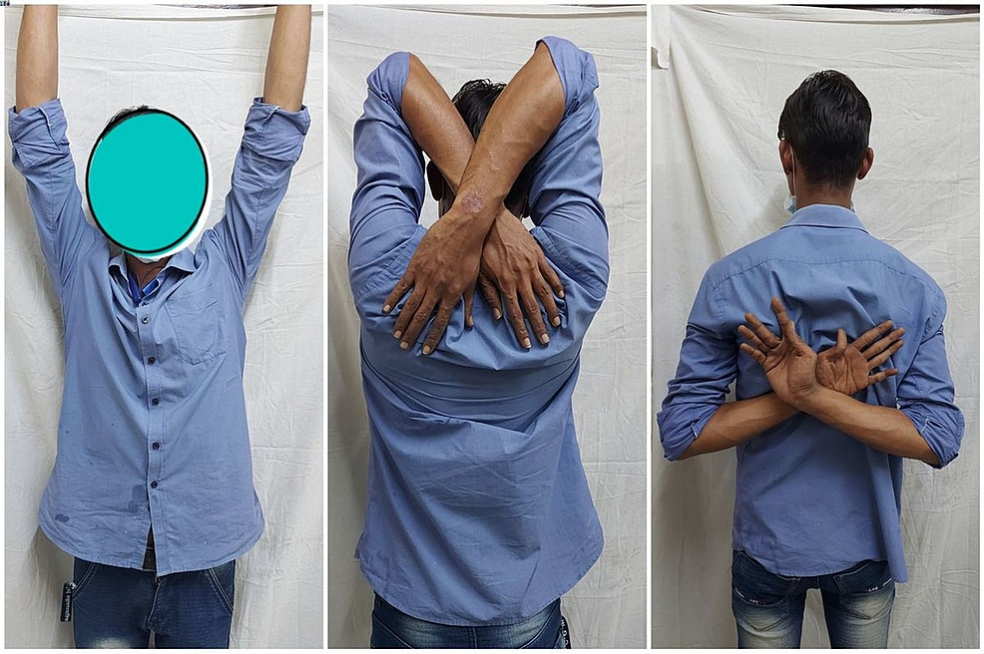
Clinical picture of a 32-year-old male showing the full range of right shoulder movement at one-year follow-up.

Our study highlights the fact that the distal radius volar plate is an excellent alternative to the hook plate in the treatment of unstable lateral third clavicle fractures. The decrease in pain and improved functional outcome stresses the fact that the volar locking plate is the recent most advancement in the fracture fixation of Neer’s type ll fractures (Figures 8, 9, 10). The fracture unites earlier in cases managed by distal radius compared to hook plate. The complications like impingement, subacromial osteolysis, and rotator cuff injuries are more with the hook plate. The anatomic configuration of the volar plate allows the implant to be in-situ with no potential complications. Our study has the largest sample size and follow-up of cases in the literature comparing the outcome of these operative modalities in the treatment of lateral end clavicle fracture. Our study has a few limitations. One, the sample size needs to be larger. Second, the mean follow-up is of 20 months and a longer follow-up is required to formulate the treatment protocol of lateral clavicle fractures.

## Conclusions

The distal radius volar plate is the recent internal fixation technique to manage unstable lateral end clavicle fractures. The volar plate restores the functional range without necessitating implant removal. Our study proposes that distal radius volar plate is an alternate superior fixation technique for the management of unstable Neer’s type II fractures.
